# Biocellulose-based hydrogel dressing as a strategy for the management of chronic arterial wounds

**DOI:** 10.1590/acb392924

**Published:** 2024-07-01

**Authors:** Carolina Magro Barreiros de Moraes, Arthur Mestriner Bassanelli, Lenize da Silva Rodrigues, Hernane da Silva Barud, Marina de Lima Fontes, Pedro Luiz Toledo de Arruda Lourenção, Meire Cristina Novelli e Castro, Matheus Bertanha

**Affiliations:** 1 Universidade Estadual Paulista – Hospital Clinics of the Faculty of Medicine of Botucatu - Department of Surgery and Orthopedics – Botucatu (SP), Brazil.; 2 Centro Universitário de Araraquara – Biopolymers and Biomaterials Laboratory – Araraquara (SP), Brazil.

**Keywords:** Peripheral Arterial Disease, Atherosclerosis, Wounds and Injuries, Wound Healing

## Abstract

**Purpose:**

To evaluate using a biocellulose-based hydrogel as an adjuvant in the healing process of arterial ulcers.

**Methods:**

A prospective single group quasi-experimental study was carried out with chronic lower limb arterial ulcer patients. These patients received biocellulose-based hydrogel dressings and outpatient guidance on dressing and periodic reassessments. The primary outcomes were the ulcer-healing rate and product safety, which were assessed by ulcer area measured in photographic records of pre-treatment and posttreatment after 7, 30, and 60 days. Secondary outcomes were related to clinical assessment by the quality-of-life scores (SF-36 and EQ-5D) and pain, evaluated by the visual analogue scale (VAS).

**Results:**

Seventeen participants were included, and one of them was excluded. Six patients (37%) had complete wound healing, and all patients had a significant reduction in the ulcer area during follow-up (233.6mm^2^ versus 2.7mm^2^) and reduction on the score PUSH 3.0 (p < 0.0001). The analysis of the SF-36 and EQ-5D questionnaires showed a statistically significant improvement in almost all parameters analyzed and with a reduction of pain assessed by the VAS.

**Conclusions:**

The biocellulose-based hydrogel was safe and showed a good perspective to promoting the necessary conditions to facilitate partial or complete healing of chronic arterial ulcers within a 60-day follow-up. Quality of life and pain were positively affected by the treatment.

## Introduction

The cases of arterial ulcers in patients with peripheral arterial disease (PAD) are approximately 20% of lower limb ulcers related to arterial insufficiency, and approximately 5 to 10% of arterial ulcers are related to arterial ischemia. The incidence of arterial ulcers has a significant impact on public health, demanding resources, and generating frustration among health professionals and patients, resulting in a deterioration in the latter’s quality of life and loss of working days^1^.

Peripheral arterial disease (PAD) is considered a chronic and progressive vascular disease, that commonly coexists with systemic atherosclerosis due to generalized endothelial dysfunction and inflammation, associated with an abnormal metabolic state^2^^-^ 9. It has a strong correlation with systemic arterial hypertension, smoking, diabetes mellitus, and hyperlipidemia, among others.3,10 PAD mainly affects the lower limbs, with an estimated frequency of 10% to 12% in the adult population and 20% in the population over 75 years old, with a predominance of males^8^^-^ 13.

Peripheral arterial disease (PAD) clinically manifests with intermittent claudication, pathognomonic symptom, pulses absence, cyanosis, pallor, fragile skin, brittle nails, and absence of hair growth^14^, At the end of the disease stability cycle, critical limb ischemia (CLI) can cause extreme walking limitation, pain at rest, ulcers, or gangrenes^15^^-^ 17. Arterial insufficiency causes a reduction in the delivery of tissue oxygen and nutrients leading to ischemia and finally ulceration^18^. Many patients, even under the best surgical treatment, persist with difficult healing ulcers (Fontaine IV, Rutherford 5 and 6), resulting from spontaneous injuries, traumas, or minor amputations, which could become chronic^19^^-^ 23.

In general, lower extremity ulcerations affect up to 49 million people annually across the globe, with 1.0 to 1.8% of cumulative lifetime risk, and is responsible for significant increases in morbidity, mortality, and high cost to health care systems worldwide^18^. The estimated incidence of CLI is 500 to 1,000 lower limbs affected per million inhabitants per year. Among these patients, the primary amputation rate ranges from 10 to 40%^8^. However, the epidemiologic data are heterogênic and limited in the scientific literature^24^. Arterial revascularization of the affected limb is mandatory in CLI, either by conventional surgeries with bypasses or by endovascular techniques^20^. In cases where revascularization is not possible, either because of the patient’s clinical condition or due to lack of arterial flow, or when revascularization is insufficient, or when there is a therapeutic failure, chronic arterial ulcers are present in approximately 22% of cases, requiring dressings for long periods^25^^,^ 26.

Although there is a wide variety of products for dressings, a single product does not have the characteristics for all types of ulcers, raising the hypothesis that the bacterial biocellulose-based hydrogel can act positively as an autolytic debriding agent and provide an organizational structure for tissue regeneration^27^^-^ 39. Biocellulose-based hydrogel (HB) does not contain lignin, hemicellulose, and pectin that require treatment to remove, neither contain components of animal origin, therefore, it does not stimulate allergic reactions. It is produced by the bacteria Gluconacetobacter xylinum^33^^,^ 36 ^,^ 39. Its morphological structure consists of nanofibers organized in a three-dimensional network, which provide unique physical and mechanical properties, high crystallinity (60-80%), and high hydrophilicity, biocellulose-based hydrogel (HB) is capable of absorbing more than 100 times its mass in water. Since it is highly porous, it confers the characteristic of being permeable^36^. Biocellulose-based hydrogel has a high purity, as the structure is a viable matrix to assist in the treatment of dermal injuries and has been used as a temporary replacement for skin, burns, ulcers, grafts, as a cover for wounds and to assist in dermal abrasions^36^. Therefore, the objective of this study was to evaluate biocellulose-based hydrogel as a strategy for the management of chronic arterial wounds, verifying its safety and potential benefits.

## Methods

### Ethic

The study was conducted in accordance with the international ethical standards of the Declaration of Helsinki and approved by the local research ethics committee with registration number 20297019.1.0000.5411. Written informed consent was obtained from all individual participants prior to their inclusion in the study, and they were allowed to withdraw from the trial at any time.

### Study design and patients

A phase I/II clinical trial, non-randomized, single-arm was performed in a single center (São Paulo State University, School of Medicine, Botucatu., Brazil). All participants were outpatients with chronic, clean arterial ulcers followed in routine basis by the Vascular Surgery team.

### Eligibility Criteria

#### Inclusion criteria

- Both genders, aged between 18 and 90 years;
- PAD confirmed by arteriography or vascular Doppler ultrasonography, which had received the best possible revascularization treatment;
- Have a foot or leg ulcer (distal third) with area of at least 1cm^2^ and a maximum of 3 ulcers on the foot or leg (distal third) totaling up to 10cm^2^ in area;
- PAD and CLI graded as Fontaine IV and Rutherford 5 or 6;
- Drug treatment for PAD (antiplatelet aggregation) and for comorbidities;
- Availability to attend medical appointments;
- Consent and sign the Informed Consent Form (ICF).

#### Exclusion criteria

- Pregnancy or puerperium;
- Have an ulcer healed during the screening period;
- Have signs of systemic or active infection in the ulcer (cellulitis, fasciitis or osteomyelitis);
- Areas of non-debrided periulcer gangrene;
- Be allergic to the product involved in this study (biocellulose-based hydrogel);
- Having had amputation at the level of the thigh in the limb to be studied.

### Intervention

After the patients signed the ICF, at the time of inclusion, ulcers with fibrin or minimal devitalized tissues were carefully debrided with a number 15 scalpel blade. Patients with necrosis or infected ulcers were not included. The dressing was applied after cleaning the ulcer with moistened gauze with 0.9% saline solution and a thin layer of biocellulose-based hydrogel (BioSmart Nanotechnology Ltda, Araraquara, Brazil, belonging to the Seven Group) over the gauze until the ulcer was covered. A secondary dressing with dry gauze and crepe bandage was applied over the primary dressing. Dressing exchanges were recommended every 12 hours after cleaning the site with 0.9% saline solution and covering them with sterile gauze and crepe straps until the ulcer healed, or for a maximum of 60 days. The patients received written and verbal instructions on how to apply the dressings. The first dressing was supervised by a nurse from the study team, who provided a tube of a biocellulose-based hydrogel. Patients were also instructed to perform dressing changes at home. In case of difficulties, they could return to the hospital at any time.

### Follow-up, assessments, procedures

#### Medical assessments

- Demographic data (includes gender, age, ethnicity, physical activity, ulcer time of evolution, and socioeconomic and educational level);
- Medical history: main comorbidities (includes peripheral arterial disease, type 2 diabetes mellitus, systemic arterial hypertension, smoking, dyslipidemia, myocardial infarction, stroke, deep vein thrombosis, and alcoholism);
- Physical examination: palpation of pulses;
- Assessment of wound healing by direct measurements and score classification: Pressure Ulcer Scale for Healing (PUSH 3-0)
- Assessment of adverse events, (better described in the safety outcomes section);
- Application of the quality-of-life questionnaire: Medical Outcomes Study 36 - Item Short-Form Health Survey (SF-36);
- Application of the pain scale: Visual Analogue Scale (VAS);
- Application of the health perception scale (Scale EQ-5D).

Observation: for the evaluation of the socioeconomic level of the families, the family income in minimum wages of the Brazilian family was used, equivalent to US$ 250.00.

#### Ulcer assessment

- Analyzing the photograph of the ulcer captures by a digital camera at four dif-ferent time points, positioned 30cm away from the lesion, observing: area (mm^2^) width and length (measured in mm)
- Ulcer evaluation with the Pressure Ulcer Scale for Healing - PUSH 3.0, which takes into account: the ratio width multiplied by length (score from zero to ten, meas-urements in centimeters); the amount of exudate (score from zero to 3) and type of tissue (score from zero to 4, healed ulcer, epithelial tissue, granulation tissue, and necrotic tissue, respectively), adding up the points for each evaluation moment^34^^,^ 35.
- Ulcer evaluation with PUSH 3.0, considering: the ratio width multiplied by length (score from zero to ten, measurements in centimeters); the amount of exudate (score from zero to three) and type of tissue (score from zero to four, healed ulcer, epithelial tissue, granulation tissue, and necrotic tissue, respectively), adding up the points for each evaluation^31^^,^ 32.

#### Dressings

After inclusion in the study (D0), all participants were instructed to repeat the dressing changes at home: clean the wound with 0.9% saline solution, cover it with a biocellu-lose-based hydrogel on gauze and a crepe strip, changing it twice a day. This method of care was repeated and reinforced at every outpatient appointment.

### Follow-up assessment

The participants were assessed and included in the study on the first visit and then followed up at 7, 30, and 60 days post-admission.

### Criteria of discontinuation

- Withdrawal of consent by the study participant;
- Lack of follow-up or non-compliance to treatment;
- Progress to major amputation on the treated limb;
- Unfavorable clinical evolution, such as the presence of serious infection or sepsis during the study.

### Outcome

#### Safety endpoint

Occurrence of adverse events related or not to the use of the product:

- Major adverse events: death, acute myocardial infarction (AMI), stroke, thromboembolic events such as deep vein thrombosis (DVT) or pulmonary embolism (PE), major amputations, systemic infections, and severe allergic reactions;
- Minor adverse events: local allergies, local infections, bleeding, worsening of pain, increased wound area.

#### Efficacy endpoint

The primary efficacy endpoint was complete ulcer healing (complete epithelization). concluding the evaluation period at 60 days of treatment. The evaluation was done on outpatient return (7, 30, and 60 days), as previously described.

#### Secondary endpoints

- Qualitative evaluation of the ulcers by direct measurements and by PUSH 3-0 score;
- Improvement in quality of life assessed by the SF-36 scale;
- Decrease in pain assessed by the VAS Scale;
- Improvement in the level of health perception assessed by the EQ-5D scale.

### Method for the qualitative and quantitative analysis of the ulcer area

In order to evaluate the quantitative results of the healing rate, photographs of the lesion were analyzed on a computer screen, by measuring the diameter in mm^2^, at the different appointments during follow-up, using the “freehand selections” tool of the ImageJ™ software. The results were given in pixels^2^ and converted into mm^2^ with the aid of the standard measurement of a ruler placed close to the ulcer. Each image was independently evaluated by two professionals experienced in ulcer assessment, and the results were presented as the arithmetic mean of the results obtained.

### Statistical analysis

Descriptive statistics of data with frequencies and percentages for qualitative variables were carried out. The Shapiro-Wilk normality test was used to assess the data distribution parameter. For quantitative and explanatory variables between points in time, with para-metric distribution, mean and standard deviation were calculated, followed by paired *t*-test, and for categorical data, the chi-square test was used. For single-sample non-parametric multivariate analyses, the Friedman test was used.

## Results

### Epidemiological data

**Figure 1:**
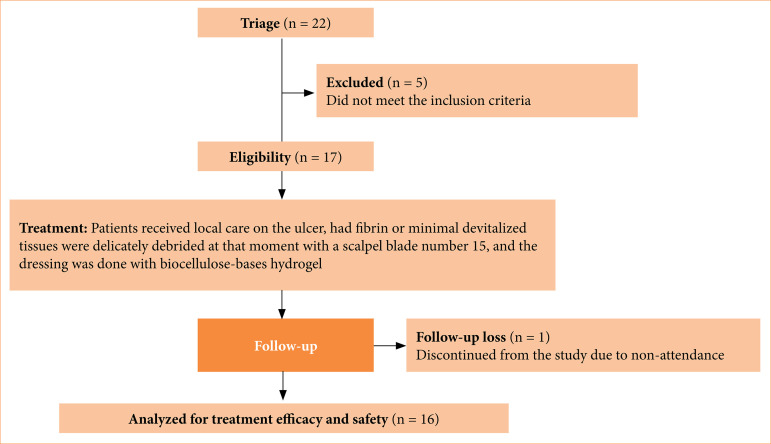
Participant selection flowchart.

Twenty-two patients with wounds were screened for the study, and 17 patients with PAD shallow arterial ulcers (Fig. 1), with no devitalized tissue in the wound bed and no signs of infection were included. One patient was excluded because of non-attendance to scheduled follow-ups. Were included, 81% men and 19% women with a mean age of 60 years (46 - 85 years), 94% associated with *Diabetes mellitus* Type 2 (DMT2), and 87% hypercholesterolemia (Table 1).

**Table 1:** Epidemiological and clinical data.

Variable			n (%)
Gender n. (%)	Male		12 (81)
	Female		2 (19)
Age in years (min - max)			60 (46 - 85)
Ethnicity n. (%)	Caucasian		13 (81)
	Mixed (African-Brazilian)		2 (12)
	Asian		1 (6)
	Black		0
Schooling n. (%)	Incomplete Primary School		11 (69)
	Complete Primary School		0
	Incomplete High School		1 (6)
	Complete High School		2 (19)
	Incomplete Higher Education		0
	Complete Higher Education		1 (6)
Family income less than 5 miniminum wages n. (%)			16 (100)
Peripheral arterial disease n. (%)			16 (100)
Systemic hypertension n. (%)			13 (81)
*Diabetes mellitus* Type 2 n. (%)			15 (94)
Smoking n. (%)			0 (0.0)
Former smoker n. (%)			13 (81)
Dyslipidemia n. (%)			14 (87)
Myocardial infarction n. (%)			4 (25)
Stroke n. (%)			1 (6)
Deep venous thrombosis n. (%)			0 (0.0)
Alcoholism n. (%)			2 (12)
Time existence of the ulcer, weeks in mean (sd)			19 (7.5)
Pulses n.(%)	Femoral	(4+/4+)	16 (100)
	Popliteal	(4+/4+)	10 (62)
		(3+/4+)	2 (12)
		(2+/4+)	1 (6)
		(NP/4+)	3 (19)
	Anterior tibial	(4+/4+)	2 (12)
		(3+/4+)	0 (0)
		(2+/4+)	0 (0)
		(1+/4+)	0 (0)
		(NP/4+)	14 (87)
	Posterior tibial	(4+/4+)	1 (6)
		(2+/4+)	0 (0)
		(NP/4+)	15 (94)

NP: non-palpable pulse

### Treatment assessment

#### A. Macroscopic

**Figure 2:**
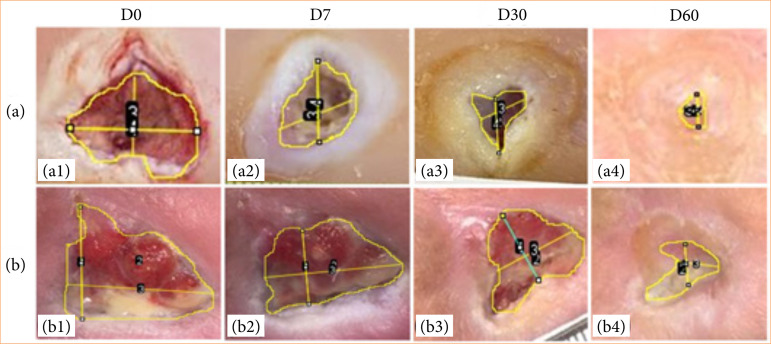
Panel of follow-up images, D0, D7, D30, and D60. a. Patient with an ulcer in the heel region **(a1)**, images from D0 to D60 **(a2 – a5)** show the measurements per-formed in ImageJ™ Software; b. Patient with an ulcer in the lateral region of the forefoot near the fifth toe **(b1)**, images from D0 to D60 **(b2 – b5)** show the measurements per-formed in ImageJ.

Figure 2 presents some examples of the results of the macroscopic assessment of ulcer healing, where we can observe a significant improvement in healing in general. The ulcers did not show marginal hyperkeratosis, tissue necrosis, or significant fibrin formation. There were no infections and all ulcers improved in terms of depth and granulation exuda-tion, proving the safety for using biocellulose-based hydrogel (Nexfill) for patients with arterial ulcers.

#### B. Quantitative assessment according to ulcers measurements

**Figure 3:**
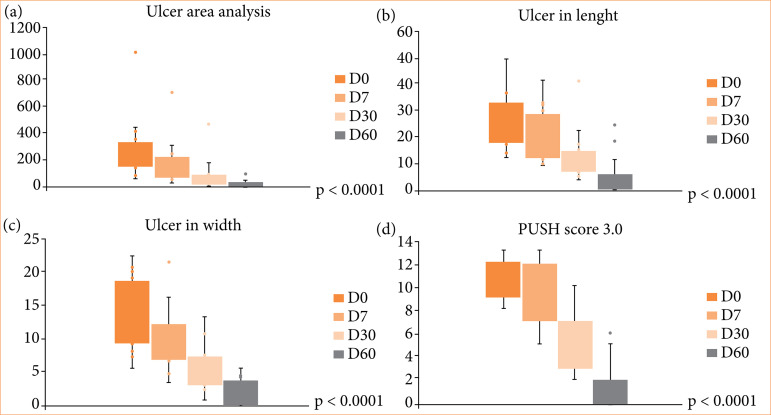
Assessment of the area of ulcer healing.

Figure 3 presents the results of the analysis of the area (Fig. 3a), length (Fig. 3b), and width of the ulcers (Fig. 3c), obtained by the ImageJ^TM^ Software. Comparing follow-ups (D0, D7, D30 and D60), there was a progressive improvement in the ulcers regarding all parameters, with statistical difference (p < 0.0001). Six patients (37%) had complete healing of the ulcers within the evaluation period of 60 days and all ulcers improved, with a mean reduction of 92% in area (Fig. 3a). There was an exponential reduction in the ul-cers over time. The first seven days (D7) showed a very significant decrease in the area of the ulcer, and this reduction was progressive until near total healing for all patients. Figure 3d shows the evolution of the ulcer according to the PUSH-3.0 classification (width x length, tissue characteristics, and secretion) over the follow-up period.

### Clinical assessment

Table 2a shows the evaluations of pain by VAS pre and post-treatment. The pain was characterized as mild by most patients (62.5%) at the inclusion moment, but for the majority of patients at the end of treatment, there was no pain (81.25%).

Table 2b presents the data from the evaluation made through the EQ-5D health questionnaire applied pre-treatment and post-treatment. The standardized measure of health-related quality of life through the EQ-5D questionnaire showed no significant variation during the 60-day medical follow-up (p = 0.050). However, specificallyin health status evaluation, we noticed that the participants reported improvement in relation to the initial status (p = 0.029).

**Table 2:** Pain assessment (VAS) and health perception (EQ5D).

(a) Results of pain analysis using the visual analogue scale (VAS)				
Moment of assessment		D0	D60	p
Pain quantification as per VAS * n. (%)	0 - 2 (mild pain)	5 (33)	13 (87)	< 0.001
	3 - 7 (moderate pain)	10 (67)	2 (13)	0.021
**(b) EQ5D assessment of health status perception**				
**Moment of assessment**		**D0**	**D60**	**p**
EQ-5D * mean (sd)		10.7 (3.9)	8.3 (3.5)	0.050
Assessment of health perception * mean (sd)		74.9 (19.6)	88.1 (9.7)	0.029

VAS = visual analogue scale; D0 = pre-treatment; D60 = 60 days after beginning treatment;

* Statistical analysis by chi-square test;

EQ-5D = EuroQol group; D0 = pre-treatment; D60 = 60 days after beginning treatment; sd = standard deviation;

* Statistical analysis by paired t-test.

Table 3 shows the results of the evaluation of the SF36 quality-of-life score, between the initial and final moments of the study. Only the mental health score and the social aspect did not have statistically significant differences, and all other relevant aspects improved with the treatment.

**Table 3:** Comparison of the SF36 score to evaluate quality-of-life between the initial and final moments.

Moment of assessment*	D0 n (sd)	D60 n (sd)	p
Funcional capacity	35.0 (32.5)	64.7 (33.3)	0.049
Limitation due to physical aspects	35.0 (32.5)	67.2 (31.3)	0.022
Pain	39.4 (7.7)	47.5 (4.5)	0.001
Vitality	54.1 (18.3)	70.3 (13.1)	0.007
General health status	67.8 (21.3)	51.6 (9.0)	0.008
Emotional aspects	43.7 (23.5)	70.8 (24.0)	0.002
Mental health	57.7 (17.5)	66.7 (16.2)	0.141
Social aspect	49.2 (8.5)	51.6 (9.0)	0.454
SF36 score	47.7 (15.1)	61.3 (12.4)	0.009

SF36 = short form health survey; sd = standard deviation; D0 = pre-treatment; D60 = 60 days after beginning treatment;

* Statistical analysis by paired t-test.

## Discussion

Arterial leg ulcers develop as a result of perfusion deficit of arterial blood to the tissues, with extremities and toes being particularly affected. In cases of CLI, with no sur-gical treatment, the limbs are amenable to spontaneous or traumatic ulcers that take a long time to heal or become chronic and never heal^14^^,^ 15 ^,^ 30 ^,^ 33.

A wide variety of dressings can be used and adapted depending on the overall purpose of the treatment. The choice is usually based on dressings that can reduce pain, control exudate, and promote a suitable environment for healing^23^. According to the Brit-ish National Formulary (BNF), the main requirements of a dressing are to keep the wound moist with exudate (but not macerated), free of infection and excessive slough, free of toxic chemicals or fibers, at the optimal healing temperature, undisturbed, and at an optimal pH level.

It is known that the use of dressings and covers produces a favorable environment for healing; however, there is insufficient scientific evidence to determine the best topical agent or dressing to promote the acceleration of healing and protect ulcers from infections^26^^,^ 36 ^,^ 38. The treatment of arterial ulcers is undertaken when the underlying disease is in its terminal phase (PAD), in which the clinical management is quite complex, and the progress is often frustrating, which includes severe difficulty in wound healing.

On the other hand, overall social conditions can influence the evolution of the pa-tients. In this study, the epidemiological data of the patients show low socioeconomic conditions and low educational levels, as well as dependency on the Brazilian Public Health System (Unified Health System - SUS). This finding may have contributed to the complex clinical status and severity of the disease presentation (CLI - PAD), influencing the chronic ulcer state, which is harmed mainly regarding changing behaviors and life habits.

The biocellulose-based hydrogel was suggested in this study as it may help in re-versing the chronification mechanisms of arterial ulcers, which was confirmed in our data, particularly at the beginning of the treatment. It provided a suitable environment for gran-ulation, skin regeneration, and the exponential reduction of the ulcer area. According to the study by Maia et al (2019), the biocellulose-based hydrogel was associated with a biocellulose-based film, which provided the complete healing of ischemic wounds after 90 days, with a healing rate of 50% compared to the control group (18.2%)^39^. Data were corroborated by our study, which achieved complete healing of arterial ulcers in 37.5% of patients, after 60 days of treatment. In addition, there was a very significant improvement for all patients, including pain assessment, quality of life, and the ulcer area decreased exponentially, in D0 233.6 mm^2^ (153.5; 291.5) to D60 2.7 mm^2^ (0; 37.0)^23^^,^ 36 ^,^ 38.

Regarding the perception of health evaluated by the SF-36 and EQ-5D question-naires, an improvement was observed over time, despite the vulnerability of the studied population. This is in line with the best results presented in the scientific literature related to the topic^22^^,^ 36 ^-^ 39.

To summarize, the limitations identified in this study are the small sample and lack of comparison group.

## Conclusions

Biocellulose-based hydrogel was safe and effective in improving the healing rate of arterial ulcers in the terminal phase of the disease, after a 60-day follow-up. The rate of wound healing, improvement in the pain caused by the wound, and improvement in gen-eral health were noticed. These results were quite significant to corroborating the im-portance of dressing in the healing process of arterial ulcers and supporting the possibility of using biocellulose-based hydrogel.

## Data Availability

All survey data can be requested from the corresponding author.
